# Challenges facing vaccinators in the 21^st^ century: results from a focus group qualitative study

**DOI:** 10.1080/21645515.2019.1621147

**Published:** 2019-07-09

**Authors:** Frédérique Wiot, Jane Shirley, Anna Prugnola, Alberta Di Pasquale, Roy Philip

**Affiliations:** a GSK, Business Intelligence, Wavre, Belgium; b Cello Health Insight, Cello Health, London, UK; c GSK, Medical Affairs, Wavre, Belgium; d Graduate Entry Medical School (GEMS), University of Limerick, Limerick, Ireland and the University Hospital Limerick & University Maternity Hospital Limerick, Limerick, Ireland

**Keywords:** Immunization, life-course immunization, market research, qualitative study, vaccinator, vaccine, vaccine hesitancy, vaccination coverage

## Abstract

**Introduction:** Barriers to vaccination and the important role of healthcare professionals (HCPs) in influencing immunization decisions made by parents/patients have been well documented. Little information describes challenges that HCPs face in carrying out their role as vaccinators.

**Methods:** We conducted a focus group study asking HCPs to describe their expectations as frontline vaccinators versus the day-to-day reality they faced. Participants described challenges impacting their ability and motivation as vaccinators, and proposed key solutions to the most important challenges. A total of 75 nurses and physicians (sixteen groups of frontline vaccinators) from the United Kingdom, United States, Germany and India participated in 2 hour focus-group discussions.

**Results:** There was disconnect between how participants viewed their role in preserving population health when they started their career, and the reality of real-world practice today. Challenges experienced and reflected were similar across professional groups and countries. Low patient-level vaccine knowledge, patient miseducation, untimely vaccine information, frequently changing vaccine schedules, time pressures, lack of centralized record systems, pressure to achieve vaccination targets, and in some instances vaccine costs, all impacted the efficiency and enthusiasm of HCPs. Identified solutions by the same providers included improving patient-level information, equipping HCPs with effective information, and practical ways to reduce the vaccination burden by improved administrative processes and centralized recording coupled with delegating vaccinator roles.

**Conclusion:** This focus group gives unique insights into needs of HCPs to fulfill their role as vaccinators. Supporting and equipping vaccinators is critical to the continuing success of vaccination programs and the proposed life-course immunization strategy. (Supplementary Appendix 1)

## Introduction

Along with clean water, good nutrition and healthy sanitation practices, vaccination is the mainstay of infectious disease control.^1^ Vaccines arrive at their point of administration as a result of decisions and actions involving numerous individuals (or groups of individuals) who evaluate, recommend, procure, distribute and oversee their availability. At the helm of administration, is the vaccinator (or vaccine provider), who is the primary individual responsible for vaccination decisions.

The healthcare environment of the 21st century is markedly different from the 1940–50s’ when vaccines were first introduced for routine use in universal immunization programs.^1^ The 21st century vaccination landscape is characterized by unprecedented access to information, rising levels of vaccine hesitancy among patients and healthcare professionals (HCPs) such that vaccine hesitance has been included in the top 10 threats to global health by the World Health Organization,^2^ as well as increasing requirements for efficiencies in healthcare delivery and documentation of healthcare interventions by HCPs. In the 21st century, dozens of vaccines are available and their use in individual countries may be mandated, recommended, or optional, with large differences in the cost to patients across countries, healthcare systems and insurance plans. By contrast, many of the infectious diseases and their consequences that are targeted by vaccination are rarely seen nowadays by physicians or the general public. Vaccination schedules vary between and within countries, and within populations (ie, recommendations for individual vaccines can vary according to age or the presence of risk factors). Vaccination schedules also change constantly as new vaccines (and older vaccines with new indications) are made available, and as new information about effectiveness and safety come to hand. Vaccines undergo rigorous and continuous testing of quality, effectiveness and safety.^3,4^ Issues around vaccine supply, safety, effectiveness and scheduling can arise at any time and be rapidly disseminated across different media platforms. In this way, frontline vaccinators can find themselves in situations where their patient appears to be better informed than they are about current vaccine issues. Furthermore, low levels of knowledge has been associated with overconfidence amongst non-professionals in their own knowledge.^5^

The frontline vaccinator remains the strongest influencer of vaccine uptake by the general population,^6–10^ and is typically an HCP who is committed to vaccinating and advocating vaccination. Numerous studies documenting the causes, effects and extent of vaccine hesitancy have reiterated the need to equip and educate frontline vaccinators in order to maintain high vaccine coverage.^10^ However, less is known about how vaccinators viewed their role versus the reality of the vaccination administration experience. We conducted a qualitative study to investigate perceived gaps between the expectations of HCPs in their role as vaccinators and the reality of the world they operate in. Sixteen groups of pediatricians, general practitioners and nurses who met screening criteria to ensure that they worked actively as frontline vaccinators were interviewed. Participants were from four countries representing different healthcare, insurance and reimbursement systems; the United States (US), United Kingdom (UK), Germany and India. We asked vaccinators about the challenges they face over every part of the vaccination journey and for practical solutions that would help revitalize their role.

## Results

Sixteen groups that included a total of 75 nurse and physician vaccinators from four countries, participated in individual and focus group discussions in 2018 between October 16–27 in the US, Germany and the UK, and November 13–17 in India. In the US, 10 pediatricians, 10 general practitioners/family physicians (GPs) and 8 nurses were divided across six groups, in the UK, 10 GPs and 10 nurses were divided into four groups, in Germany, 9 pediatricians and 8 GPs were divided into four groups, and, in India 10 pediatricians were divided into two groups (Table 1).

**Table 1.** Number of participants and focus group discussions.

	United States 6 groups	United Kingdom 4 groups	Germany 4 groups	India 2 groups
Pediatricians	10 participants	–	9 participants	10 participants
General Practitioners	10 participants	10 participants	8 participants	–
Nurses	8 participants	10 participants	–	–

### The role of HCPs as vaccinators: expectations versus reality

**Figure 1.**
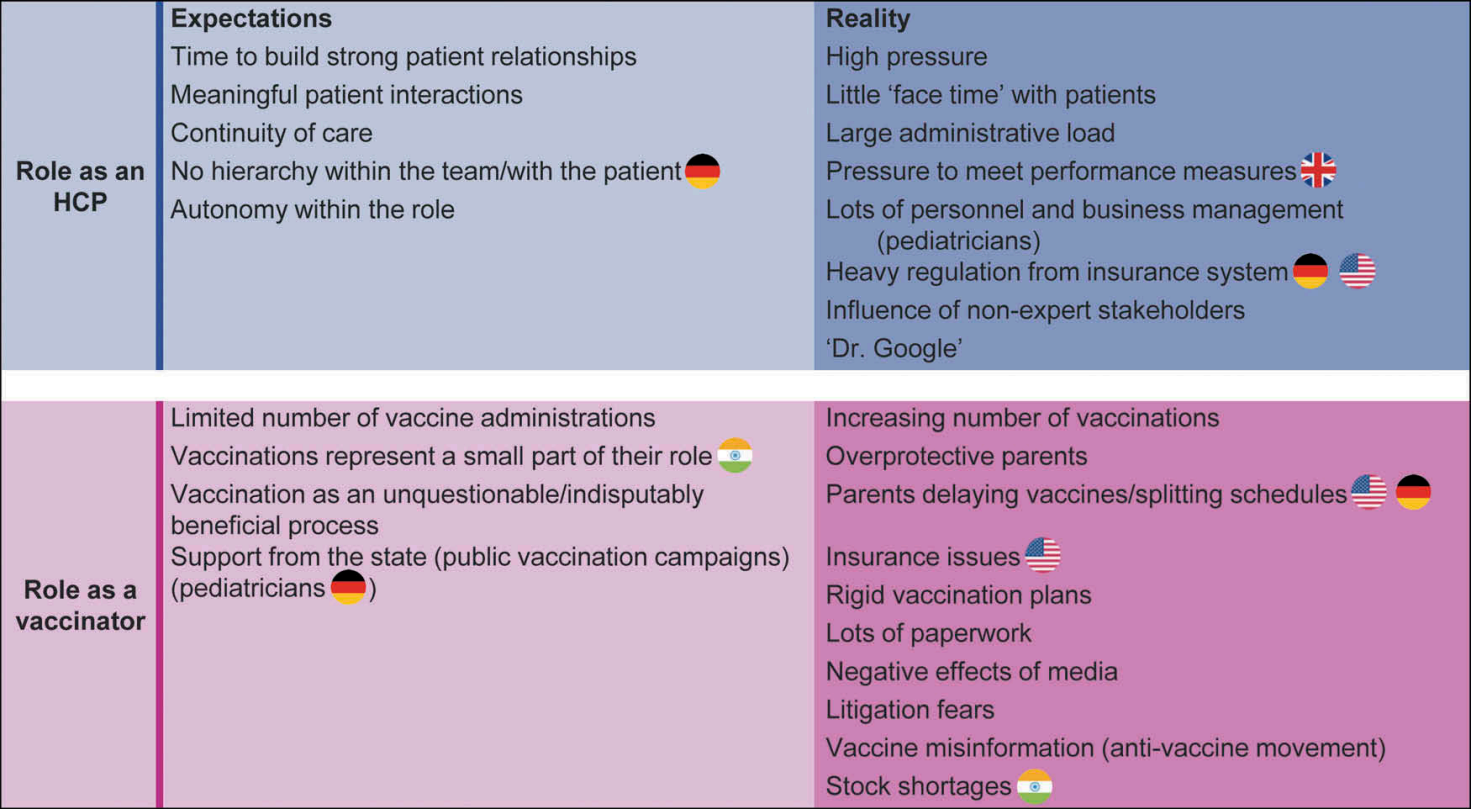
The role of vaccinator: expectations versus reality.

**Figure 2.**
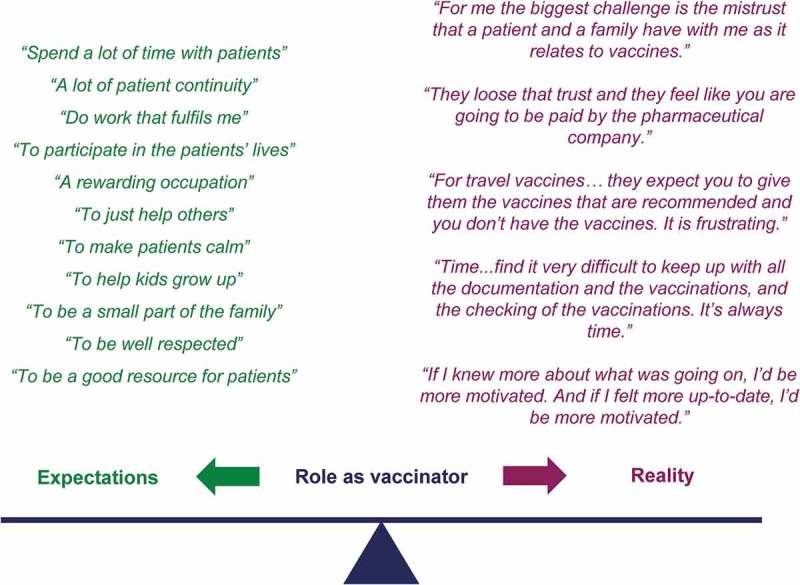
Healthcare provider opinions about the expectations versus reality of the vaccinator role.

There was a significant disconnect between how participants viewed their role as HCPs, and more specifically as vaccinators, and the realities of real-world practice (Figure 1). The reasons underlying this disconnection were similar across the different professional groups and between individual countries. While vaccinators were expected to have meaningful encounters with patients, underwritten by continuity of care and a solid conviction by patients in the benefits of vaccination, the reality was characterized by large administrative loads, constricting influences of regulations, rigid vaccination plans, and extensive time spent educating and convincing parents to accept vaccination associated with a sense of loss of trust, as illustrated during interviews (Figure 2). HCPs expected immunization to be a well-supported directive process, but the evolving reality is a ‘battle’ against vaccine misinformation and substantial paperwork. HCPs expressed feelings of guilt, frustration and disillusionment due to the loss of patient centricity and the feeling that they should be doing more for their patients. HCPs reported that unexpectedly, vaccination episodes in clinical practice were taking time away from other priorities because of the increasing number of vaccinations available, and the increasing time spent convincing patients of their value.

#### Country-specific findings on the role of HCPs

In addition to the common findings stated above, pediatricians in Germany expected to receive support from the state in terms of public vaccination campaigns. The reality was heavy regulation from the insurance system and issues with parents delaying vaccines or splitting schedules. These challenges were also highlighted in the US. In the UK, pressure to meet performance targets was highlighted as a key challenge. In India, vaccine shortages and cost of vaccines were highlighted as an additional challenge.

### Challenges faced as a vaccinator and solutions proposed by HCPs

**Figure 3.**
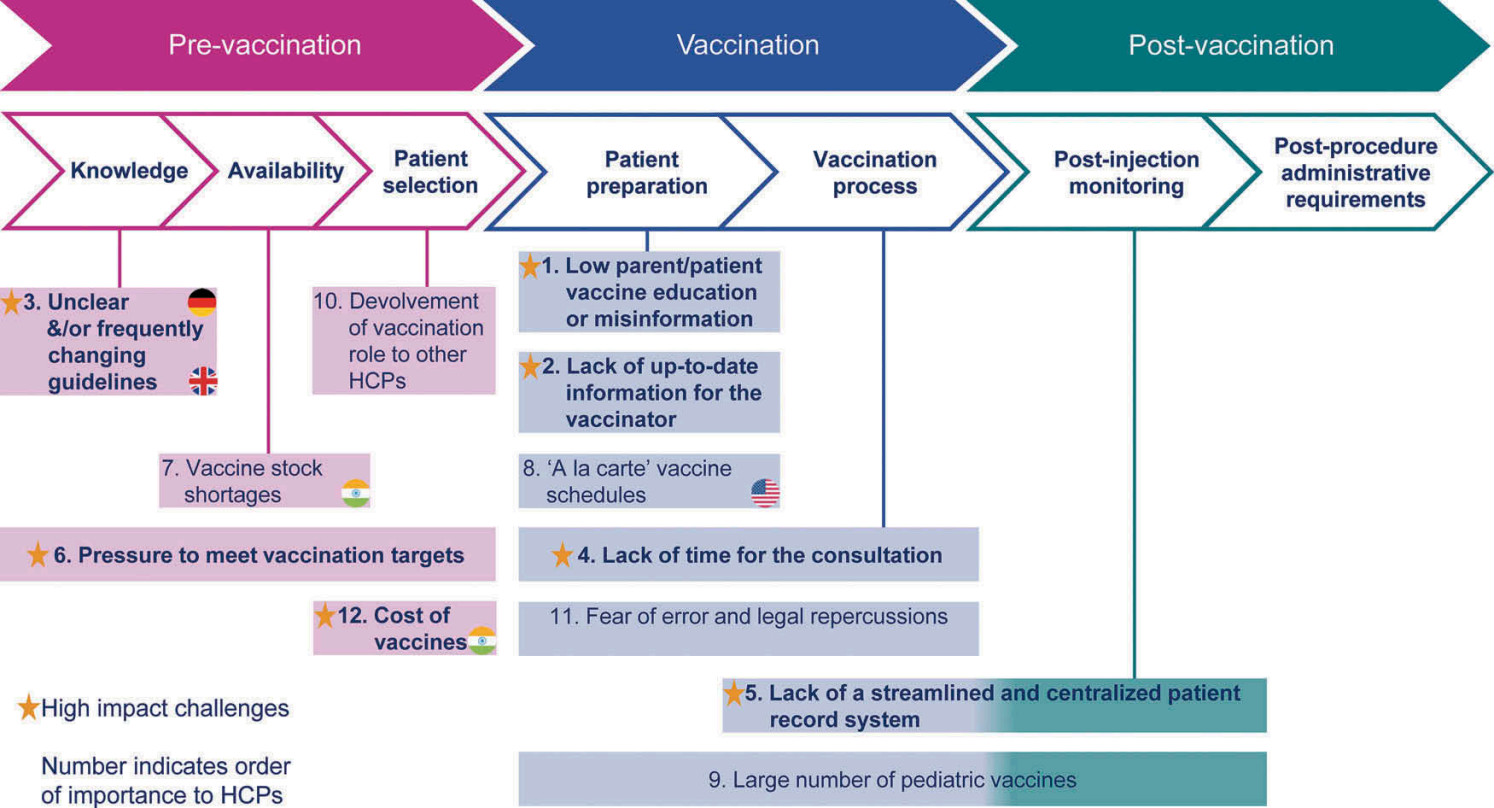
Challenges faced by vaccinators at each stage of the vaccination process.

**Figure 4.**
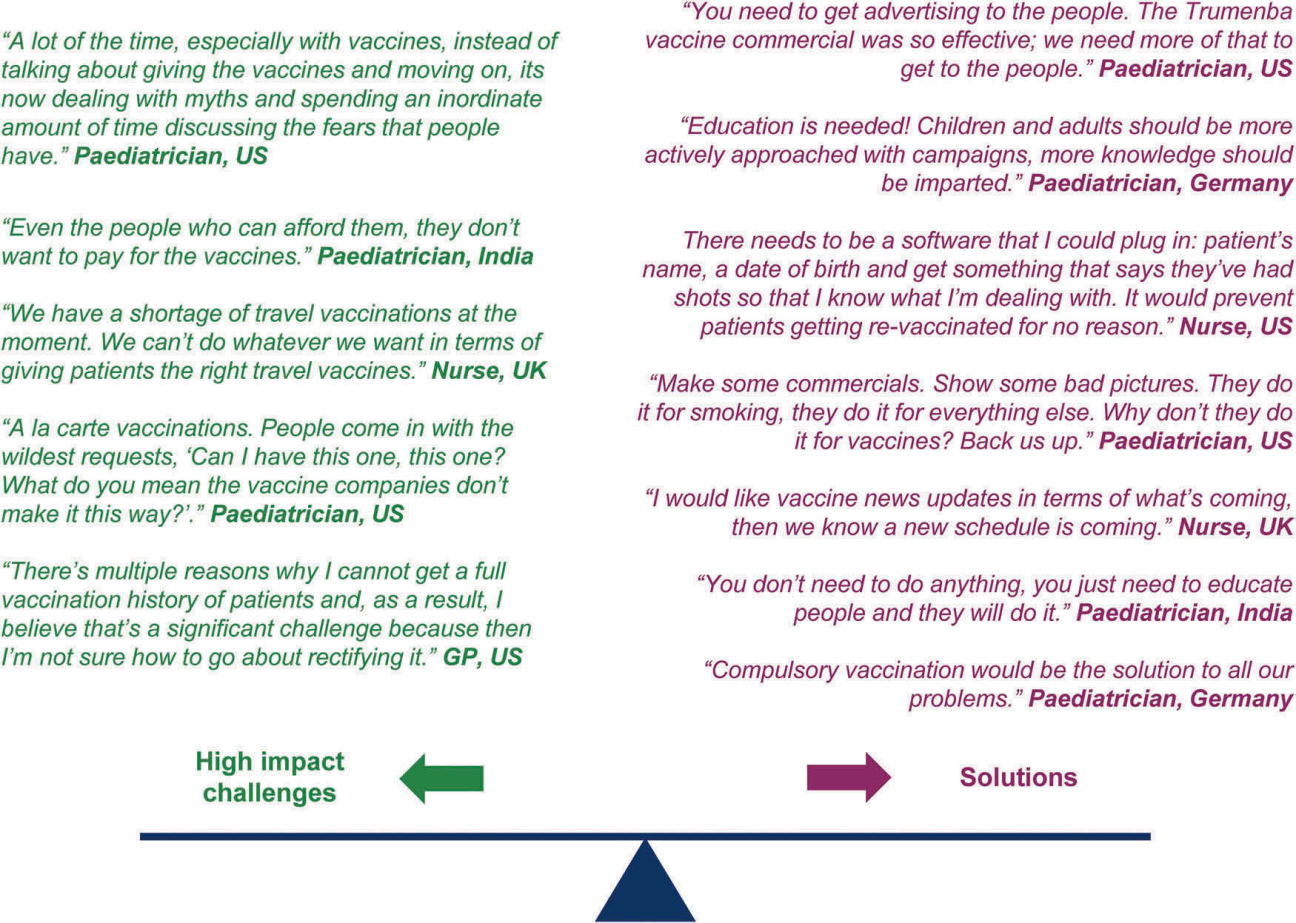
Challenges and some ideas to solve the issues expressed by healthcare providers.

Vaccinators identified challenges during all stages of the vaccination journey – from the background period prior to seeing a patient, during the pre-vaccination consultation phase, administration process, and during the period after vaccination. The overall and country-specific challenges are described below and shown in Figure 3. These challenges had varying degrees of impact on the ability of HCPs to be efficient and enthusiastic vaccinators. Potential solutions identified by HCPs for some of these challenges are provided below (Figure 4).

#### Challenges faced by all countries

During the pre-vaccination phase, **“vaccination targets and pressure to achieve them”** was identified as a high impact challenge. It detracted at times from HCPs’ ability to provide expected standards of care, particularly with respect to reaching targets for seasonal influenza and pneumococcal vaccines for adults. HCPs indicated that this led to feelings of powerlessness, reduced flexibility when consultations were focused on reaching targets, and was seen as ultimately taking away from provision of holistic care.

There is a growing move in some countries to **“devolve vaccination responsibilities from physicians and nurses to pharmacists or non-medically qualified persons”**. HCPs acknowledged that sharing the vaccination load could reduce practice workloads. On the other hand, this low-impact challenge during the pre-vaccination phase poses potential risks, identified particularly by UK vaccinators (see below).

During the vaccination and post-vaccination phases, the number one high-impact challenge in all countries, was the problem of **“little knowledge or misinformation about vaccines by parents/patients”** (Figure 3). Patients/public presenting with a lot of information and misinformation available via media (especially the Internet) required substantial time to discuss/attempt to correct or reverse misinformation and convince patients/parents to agree to vaccination. HCPs believed that this time would be better spent seeing to other patient needs. The potential solution proposed by HCPs was a multi-faceted patient/public education approach to challenge vaccine misinformation while providing patients with the information they need to make informed decisions (Box 1).

**Box 1.** Solution 1: A multi-faceted patient education approach to challenge vaccine misinformation while providing patients with the information they need to make informed decisions, including:

● Providing general information via patient-directed information leaflets at each vaccination
● Surgery waiting room videos on the importance of vaccines and on local vaccine schedules
● Educating new parents during prenatal and antenatal contacts (seen as particularly relevant in Germany and the UK)
● Inclusion of vaccine education in the school health syllabus (seen as particularly relevant in India)
● Use of positive public vaccination health campaigns
● Television and radio campaigns advocating the importance of public immunization.

HCPs and patients have an expectation that **“sufficient time will be available to discuss parent/patient questions and concerns about vaccinations”**. However, consultation times are usually fixed and often restricted to 10–15 minutes, and ‘time’ was identified as a frequent and recurring high-impact vaccination phase challenge for HCPs, as more administrative (including vaccine-related administrative requirements) and logistical factors impacted on the consultation itself. Consultation times are often based on the time needed to address one health concern. When the patient record shows that one or more vaccines are also required, HCPs must assess the need to vaccinate at that appointment based on risk (exposure to a preventable disease and likelihood of returning for a second visit). HCPs expressed that time constraints and pressures limited their effectiveness to provide best patient care.

Another high-impact vaccination/post-vaccination phase challenge occurs **“when patients move within a country or to another country with incomplete records”**. The absence of centralized patient records leads to unnecessary use of consultation time to determine the vaccination history, and wasteful use of vaccines should patients need to be re-vaccinated “just in case”. HCPs also noted that the mandatory processes by which vaccinations must be recorded are complicated, and that the vaccine administration itself was short in comparison to the time required to record their administration. HCPs proposed to reduce the logistic and practical burden via solutions that improve the recording and sharing of patient record information and administration processes (Box 2).

**Box 2.** Solution 2: Solutions to improve the recording and sharing of patient record information and streamline administration processes.

● Streamlined and centralized patient vaccination record system
● Make software available to conduct patient reminders/patient recall to complete required vaccination schedules (seen as particularly relevant in the US)
● Simplify communication between different HCPs involved in vaccines, and improve the timeliness of these communications
● Development and availability of more combination vaccines to reduce the number of injections required for infants and children
● Increased use of needle-less systems to ease the logistics of vaccine administration.

A medium-impact challenge during the vaccination/post-vaccination phases in all countries was identified by pediatricians and/or nurses commenting on the **“ever-increasing number of vaccinations required during childhood and adolescence”**. The frequency of vaccination conflicts with parents’ reluctance to “inflict pain” during the first years of life, and requires additional explanation and reassurance from HCPs. Parent reluctance to administer multiple injections at the same visit increases the logistical challenge for HCPs in ensuring that pediatric patients receive all their vaccines in a timely manner. Patient records need to be up to date and clear when vaccinations are due, in order to remind parents at other appointments. This process is further complicated if the patient is vaccinated by another HCP and this administration is not included on medical records.

A low-impact challenge during the vaccination/post-vaccination phases in all countries, was due to a growing demand from patients for a ‘human touch’ during their consultations, combined with a reduced tolerance for error. As a result, HCPs are increasingly encouraging and administering vaccines more cautiously in order to protect themselves from potential **“legal repercussions in the event of an unexpected outcome”**. The threat of legal proceedings negatively impacts on their ability to be efficient and enthusiastic vaccinators.

#### Challenges in Germany

In the pre-vaccination phase, high impact challenges identified were **“uncertainty surrounding current immunization guidelines”**, as well as **“frequent changes to the immunization schedule”**, often occurring at short notice, creating logistical and practical challenges in terms of identifying which patients should be vaccinated. Some HCPs were not sure where to access the most up-to-date vaccine guidance, which added further time to the consultation. HCPs were often left to justify changes to schedules to parents/patients when someone once eligible, was no longer eligible under the new recommendations. This led patients to question the credibility of the need for vaccination. HCPs identified that they need to be confident in delivering patient care and be well equipped to answer questions and address objections about vaccinations via HCP education and more flexible guidelines (Box 3).

**Box 3.** Solution 3: Ensure HCPs are confident in delivering patient care and feel well equipped to answer patient questions and address objections via HCP education and more flexible guidelines.

● Access to transparent, reliable and timely vaccine information/education for HCPs, via:
○ Representatives (an important information source in the US, the UK and Germany)
○ Podcasts
○ Webinars (seen as particularly relevant in the US and the UK)
○ Forums for online peer discussions
○ Scientific congress presence by vaccine manufacturers with symposia and masterclasses (seen as particularly relevant in Germany)
○ E-mails (seen as less favorable by all participants).
● Provision of clear but flexible guidelines and targets (including insurance requirements in the US) that are compatible with the real-world healthcare system. That is, affording HCPs more autonomy about which patients should or should not receive specific vaccines.

**“Recurring vaccine stock shortages”** was a medium-impact pre-vaccination challenge. Vaccine shortages require HCPs to risk-stratify individual patients, and reduces their ability to provide all-round patient care. Explaining to patients that they are of lower risk than others, and therefore not able to be vaccinated during the shortage undermines the importance of vaccination, which can be counter-productive in an environment where patients are not always advocates of vaccination.

A high-impact challenge during the vaccination and post-vaccination phases included **“poor timing of up-to-date vaccine information provided to HCPs”**. GPs felt they were not informed quickly enough about current vaccine issues. This included the availability and recommendations for new vaccines, changes to existing schedules, the most recent information around side-effects, and new information arising from research studies. HCPs felt they were at a disadvantage when faced with patient questions, and were often in the situation where the parent/patient appeared more informed (frequently incorrectly) via the media than the HCPs conventionally informed via official/professional channels.

A medium-impact challenge during the vaccination/post-vaccination phase was due to increasing discussions with **“parents trying to design their own vaccine schedule”** which may go against recommendations and insurance requirements, thus restricting HCPs’ capacity to immunize their patients appropriately.

#### Challenges in the UK

As was the case in Germany, in the pre-vaccination phase in the UK, high impact challenges identified were **“uncertainty surrounding current immunization guidelines”**, as well as **“frequent, often short notice, changes to the immunization schedule”**. As in Germany, this led to increased consultation time and discussions with patients, with HCPs identifying solutions in Box 3.

*“What challenges me most is trying to make head or tail of the shingles vaccination schedule. You’ve got who gets a turn, and in which year, and why it has been planned out so ridiculously to have different age groups every year? … the whole vaccine schedule changes so rapidly from year to year.”* UK nurse.

**“Recurring vaccine stock shortages”** was a medium-impact pre-vaccination challenge that led to risk-stratification and selection of patients for vaccination, undermining the importance of vaccination for the patient. Stock shortages currently impact travel vaccines, but shortages of generally recommended vaccines have also occurred.

A low-impact pre-vaccination challenge was the **“devolvement/shared vaccination role with other HCPs”**. UK HCPs highlighted issues such as breaks in communication between these other vaccinators and GPs resulting in a loss of continuity of care and incomplete patient records. Concerns were expressed by nurse vaccinators that other providers (such as pharmacists) would not adhere to the same care practices nor engage in appropriate clinically relevant discussions with patients. HCPs also questioned the logistics of managing vaccination targets if vaccine responsibilities were shared. Box 4 outlines potential solutions to obtain the most benefit from sharing the vaccinator role.

**Box 4.** Solution 4: Enable the sharing and development of the vaccination role across HCPs types to free up consultation time mainly through.

● Training of healthcare assistants to perform and provide information on vaccines to patients, to relieve general practitioners and pediatricians of this role (seen as particularly relevant in the UK and Germany)
● Devolvement of a vaccination role to pharmacists (not favored by nurses)
● Delegate administration of all travel vaccines to independent travel vaccine centers.
Other lower priority suggestions:
● Put in place insurance company or government-based reward systems to encourage vaccinations (only considered important in Germany)
● Mandate the use of vaccines (seen as less relevant in the UK and of most relevance in India)
● Greater government involvement in vaccine campaigns to subsidize the cost of vaccines (only considered important in India).

During the vaccination and post-vaccination phases, **“rapid provision of up-to-date vaccine information (e.g., new recommendations, schedules, side-effects) to HCPs”** was a high-impact challenge identified by GPs and nurses. This resulted in problems when dealing with patient questions that were often poorly-informed through the media.

#### Challenges in the US

A medium-impact challenge during the vaccination/post-vaccination phase was due to **“parents increasingly trying to design their own vaccine schedule”** based on decisions made from their own research. These ‘*a la carte’* schedules may conflict with vaccination recommendations or insurance requirements and they limit the ability of HCPs to effectively immunize their patients. Discussions about parent-designed schedules further extend the vaccination process.

#### Challenges in India

In the pre-vaccination phase in India, **“uncertainty surrounding current immunization guidelines”** was a high impact challenge, as was the **“cost of vaccines”**. Some of the more recently implemented and more expensive vaccines, such as pneumococcal conjugate vaccines, are recommended but not provided for free. Given the low incomes of a large proportion of families in India, the affordability of vaccines is a challenge for many. Furthermore, a general lack of knowledge of the disease causes patients to question the need for vaccines, especially given the (perceived) high cost.

**“Recurring vaccine stock shortages”** was a medium-impact pre-vaccination challenge that led to vaccination of select patients according to risk. This decreases the value of vaccination in patients’ eyes.

During the vaccination and post-vaccination phases, the number one high-impact challenge faced by all countries, was the problem of **“little knowledge or misinformation about vaccines by parents/patients”**. In India in particular, HCPs were faced with a lack of general understanding about the purpose of vaccines among sections of the population.

## Discussion

Across all countries, HCPs expected, in their role as vaccinators, to deal with a limited number of vaccines and to have the trust of patients who accept the benefits of vaccination. The reality, however, is that there are an increasing number of vaccines with rigid vaccination plans and a lot of paperwork. The HCPs often have to deal with misinformed anxious parents that question the value of vaccination due to negative press. Specifically, in Germany and the US, parents attempt to delay or modify vaccine schedules while insurance companies exert additional pressure through regulations. In India, vaccine shortages and vaccine cost are issues, as are travel vaccine shortages in the UK. In the 21st century, patient education/miseducation, often obtained through the Internet and social media channels, has emerged as one of the most important factors influencing vaccination readiness in the real-world setting. In a survey of US pediatricians and GPs published in 1994, vaccine cost and lack of insurance coverage were among the most commonly identified barriers to vaccination.^11^ According to our survey, the issue of vaccine cost continues to dominate in India where more expensive pediatric, new maternal and adult vaccines are not yet considered affordable by governments, and where substantial differences exist in the vaccines supplied through the public versus the private systems. In the US, UK and Germany, the need to address patient misinformation coupled with associated perceptions of loss of trust, contribute to a wide disconnect between the role as vaccinator versus the reality of clinical practice. In parallel, new vaccines, new indications, changes to schedules and rapid global dissemination of vaccine information have left some HCPs feeling poorly informed and ill-equipped to address questions, concerns and misinformation coming from parents/patients. These feeling are compounded by frustration and feelings of inadequacy in being able to provide holistic patient care within the timeframes available, and frustration with the administrative processes involved.

There is a great deal of literature about vaccine confidence or hesitancy among HCPs and how HCP confidence correlates with coverage.^6,10,12–16^ Other studies examining communication methods have highlighted parents’ needs for impartial discussions about vaccines from HCPs, using patient-tailored information.^17–19^ Much less is written about the challenges HCPs themselves face in undertaking this role. In our study, HCPs identified that patients’ low vaccine education/miseducation, untimely and/or unclear vaccine information, frequently changing vaccine schedules, lack of time in consultations, lack of central patient record systems, pressure to achieve vaccination targets, and vaccine cost, all highly impacted their efficiency and enthusiasm. These findings are consistent with other studies reporting that HCPs frequently feel unprepared to discuss vaccine issues, and that they face time constraints during vaccine visits.^6^ Inability to track under-vaccinated patients and incomplete immunization records were identified as key barriers to immunization in a 1994 survey.^11^ These aspects were also raised as high impact challenges in the present study, suggesting that progress in this direction still needs to be made. Unique insights from our study are the perception among some HCPs of loss of trust from their patients with respect to vaccination, and the negative impact of complex and changing vaccination schedules on HCPs’ ability to be effective vaccinators. Trust in vaccination is not always specifically evaluated.^20^ The causes of reduced patient trust and the impact on HCPs have not, to our knowledge, been explored. The negative impact of rapidly changing and complex vaccination schedules on vaccinators has also perhaps been underestimated.^21^ Health authorities often attempt to contain costs by limiting access to subsidized vaccines to those most at risk, or by rolling out vaccination campaigns to different age groups over consecutive years. However, the frontline vaccinator is left to explain these strategies to patients, on the one hand trying to advocate vaccination, and on the other having to explain to patients/parents why one individual is eligible for vaccination, while another is not. These apparent inconsistencies potentially risk undermining vaccination as a whole.

Focus groups are a complementary tool to other scientific research that can provide nuanced insights into real-world experiences. In this study, dynamic discussions within small groups of vaccinators allowed deep insights into their perceived versus actual roles, and encouraged their expression of challenges and proposed solutions based on their experiences as real-world vaccinators. Findings from this survey may stimulate reflection on why vaccination practice is not as smooth as expected and help identify solutions that can be integrated into daily practice.

The solutions identified by HCPs centered around improving the quality of patient information, better equipping HCPs with the information they need to be effective vaccinators and vaccine advocates. As a last solution, HCPs highlighted the need to reduce the workload associated with vaccination by improving administrative processes and centralized recording, plus possibly devolving vaccinator roles. Study participants stressed that HCP and patient-focused communication and education must be seen to be ‘independent’, free from bias, and easily accessible. Solutions involving changes to national healthcare systems/processes should be approached as long-term commitments, and require substantial investment and resources to overcome potential bureaucratic and legal barriers.

Strengths of the survey are the inclusion of vaccinators from a variety of healthcare backgrounds from developed countries and a developing country. The four countries represented different healthcare systems, which increases the transferability of our insights to other settings. Limitations include the small sample size, convenience sampling methodology and the “one-off” nature of the survey.

Frontline vaccinators are the most important influencers of vaccine uptake by the general population.^6–10^ However, HCPs expectations of their role as vaccinators are not matched with reality. This experience extends across professions and countries. Education and information campaigns for patients and HCPs will address the challenges that have the highest impact on vaccinators: those of poor patient education and unclear/untimely HCP-directed information. Solutions which look to establish clear guidelines and to streamline and centralize the patient record system processes will address challenges that exist across multiple stages of the vaccination journey. Supporting and equipping vaccinators is critical to the continuing success of vaccination programs and the proposed life-course immunization strategy.

## Methods

Through focus groups, a qualitative market research survey following a social constructivism perspective was conducted and a non-probability method of convenience sampling was adopted.

### Country-specific group selection

The four study countries (US, UK, Germany and India), were selected to provide views from HCPs working in very different vaccine administration environments. In the US, vaccines are usually administered by physicians (mainly pediatricians) while nurses have a key role in terms of providing advice, guidance and preparing patients for vaccination. Non-physician HCPs, such as medical/physician assistants, nurses and pharmacists can be authorized to administer vaccines according to laws that vary at a state-by-state level.^22^ The cost of vaccines varies according to insurance status, but are free for those eligible for the Vaccines for Children Program.^23^ In the UK, vaccines recommended by the National Health Service (NHS) are free of charge to families,^24^ and vaccines are primarily administered by nurses and general practitioners. In Germany, vaccines are given by private physicians or general practitioners in their offices.^25,26^ Vaccines recommended by the German Standing Vaccination Committee (STIKO) are available free of charge through the statutory insurance policy.^24^ In India, most vaccines recommended by the Indian Academy of Pediatrics (IAP) are provided free of charge.^27^ However, some vaccines such as pneumococcal conjugate vaccines and acellular pertussis vaccines are only available through the private sector. This holds true for maternal vaccination (other than the conventional tetanus toxoid), and those vaccines recommended for adults and older age groups as part of the life-course approach. This, combined with a perception among some parents that internationally manufactured vaccines may be more effective than locally made products, means that many parents opt to be vaccinated through the private sector.

The research was conducted by an independent market research company (Cello Health Insight). Potential participants were contacted by telephone or email from databases of HCPs held by the company and their locally-based suppliers. HCPs were screened using a standardized form (Supplementary Appendix 2) to ensure the vaccinators selected from each country were representative of that role in the region, thus able to reflect frontline concerns and challenges.

To be eligible to participate, HCPs had to spend 70% of more of their time in direct patient care; have been in practice between 3 and 30 years; have administered and/or recommended/personally discussed measles-mumps-rubella/varicella and diphtheria-tetanus-pertussis-containing pediatric vaccines with patients in the last 3 months; and been involved in the administration/prescribing of vaccines or responsible for discussing vaccine options and making recommendations to adults/adolescents/children. GPs and nurses were additionally required to have recommended/personally discussed at least two adult and/or travel vaccines with patients in the last 3 months.

Participants could not have participated in vaccination-related market research in the last month. HCPs (or their family members) affiliated with any pharmaceutical company, healthcare manufacturer or market research company, were not eligible to participate. In the US, HCPs could not participate if they were a government employee or if they were licensed to prescribe medications or practice/work in a medical capacity in Vermont or Minnesota. All US participants had to be board certified or board eligible in their specialty. Half of the US participants were selected from independent practices (outpatient practice with single site), and the other half from an Integrated Delivery Network (outpatient practice with multiple sites, hospital-based practice or health systems headquarters).

A quota was set in terms of the number of participants and HCP type in each country. All participants were offered financial reimbursement for their participation in the 2 hour group discussion.

### Survey description

Two hour one-to-one and group discussions were undertaken to gain insights into the understanding HCPs have of their role as vaccinators and to identify the challenges they face in this role. All sessions were facilitated by an experienced researcher from the market research company. All participants provided written consent to participate and the study sponsor was not disclosed to participants. Anonymity of the participants was maintained during video recordings and in transcripts of the meetings.

### Analysis

Individual and focus group responses were analyzed following narrative analysis principles (including word and phrase repetitions). We conducted a detailed local language analysis of the recordings followed by a thematic analysis performed by experienced specialist healthcare researcher through a phenomenological lens. Key themes were identified and discussed to ensure consistency. The analysis was conducted in a precise, consistent, and exhaustive manner in order to guide appropriate interpretation and identification of key data points. Data were analyzed according to profession-specific and country-specific information disclosed through the survey. The study adhered to standards for reporting qualitative research (SRQR) guidelines.^28^

### Ethics approval

This was a market research activity and no ethics approval was sought.

Ethical considerations for focus groups, as for other social research methods, involve informing potential participants of the purpose of the research, and how their contribution will be used, how sensitive material and confidentiality will be handled (clarify that participants contributions will be shared with others in the group and that data will be anonymized).^29^ In this study, written consent was obtained from all participants (a screener was used to provide extensive information about the study, inform potential participants about confidentiality and anonymity of results, inform them that the sponsor was a pharmaceutical company but that the research is non-promotional, allow the potential participants to accept or decline participation at several points). The study is compliant to general data protection regulation and adheres to standards and techniques for healthcare market research EpHMRA and BHBIA guidelines. Experienced researchers facilitated all the focus groups, the sponsor was not disclosed and anonymity of participants was maintained throughout.
