# Supplementary material for: Challenges facing vaccinators in the 21st century: results from a focus group qualitative study
Source: Hum Vaccin Immunother. 2019 Jul 9;15(12):2806–15. doi: 10.1080/21645515.2019.1621147 (PMC6930098; doi:10.1080/21645515.2019.1621147)
Supplement: Supplemental Material [file khvi-15-12-1621147-s001.zip › Truth about vaccinators_SuplApp2.docx]

**ADDITIONAL QUESTIONS PRIOR TO SCREENING – US ONLY**

i) Are you currently a government employee (federal, state, local)?

**SINGLE CODE**

| Yes | 01 | **GO TO ii)** |
| --- | --- | --- |
| No | 02 | **GO TO S1** |

ii) Thank you for indicating that you are employed by the government. Regulations exist stating that an honorarium or enticement cannot be paid to government employees to participate in market research.  Your opinion is valuable to us.  Do you choose to continue and complete the survey without payment?”

**SINGLE CODE**

| Yes | 01 | **GO TO iii)** |
| --- | --- | --- |
| No | 02 | **THANK & CLOSE** |

iii) Are you licensed to prescribe medications by either of the following states:

**SINGLE CODE**

**MINESOTA**

| Yes | 01 | **THANK & CLOSE** |
| --- | --- | --- |
| No | 02 | **GO TO iv)** |

**VERMONT**

| Yes | 01 | **THANK & CLOSE** |
| --- | --- | --- |
| No | 02 | **GO TO iv)** |

iv) Do you practise or otherwise work in a medical capacity in Vermont, even for only a limited time or any sort of regular frequency – for example, one day per year, or one week every three years?

**SINGLE CODE**

| Yes | 01 | **THANK & CLOSE** |
| --- | --- | --- |
| No | 02 | **GO TO S1** |

**SCREENING QUESTIONS – ASK ALL**

S1 Can you please confirm your medical speciality?

**SINGLE CODE**

| Paediatrician **[US, India & Germany ONLY\|]** | **01** | **US: GO TO S2. GE & INDIA: GO TO S4** |
| --- | --- | --- |
| Practice Nurse / Nurse Practitioner **[US & UK ONLY]** | **02** | **US: GO TO S2. UK: GO TO S4** |
| General Practitioner **[UK ONLY]** / Family Practice / Internal Medicine **[US & Germany ONLY]** | **03** | **US: GO TO S2. UK & GE: GO TO S4** |
| Other | **04** | **THANK AND CLOSE** |

**SEE BELOW FOR QUOTAS**

S2    **ASK US ONLY:** Are you board certified or board eligible in your specialty?

**SINGLE CODE**

| Board certified | 01 | **GO TO S3** |
| --- | --- | --- |
| Board eligible | 02 | **GO TO S3** |
| Neither | **03** | **THANK AND** **CLOSE** |

S3 **ASK US ONLY:** Which of the following best describes the organization / practice where you spend most of your time?

**SINGLE CODE**

| Outpatient practice with single site | 01 | **GO TO S4** |
| --- | --- | --- |
| Outpatient practice with multiple sites | 02 | **GO TO S4** |
| Hospital based practice | 03 | **GO TO S4** |
| Health system headquarters | 04 | **GO TO S4** |
| Other | 05 | **THANK AND CLOSE** |

.

**IF CODES 01 CLASS INDEPENDENT PRACTICE**

**IF CODES 02, 03 OR 04 CLASS AS INTEGRATED DELIVERY NETWORK (IDN)**

**AIM TO RECRUIT HALF IDN AND HALF INDEPENDENT**

S4 Since completing your medical training, how many years have you been in clinical practice?

**SINGLE CODE**

| Less than 3 years | **01** | **THANK AND CLOSE** |
| --- | --- | --- |
| 3-10 years | **02** | **GO TO S5** |
| 11-20 years | **03** | **GO TO S5** |
| 21-30 years | **04** | **GO TO S5** |
| More than 30 years | **05** | **THANK AND CLOSE** |

**ENSURE A MIX OF CODES 2 – 4 IN EACH GROUP**

S5 What proportion of your time is spent in direct patient care?

**SINGLE CODE**

| Less than 70% | **01** | **THANK AND CLOSE** |
| --- | --- | --- |
| More than 70% | **02** | **GO TO S6** |

S6 Does your practice vaccinate patients on a regular basis?

**SINGLE CODE**

| Yes | **01** | **GO TO S7** |
| --- | --- | --- |
| No | **02** | **THANK & CLOSE** |
| Not sure | **03** | **THANK & CLOSE** |

S7 Which of the following activities are you personally involved with, with regards to vaccination of [children/adolescents/adults] at the setting in which you work?

| **MULTICODE** | **S7a:children /adolescent** | **S7b: adult** |  |
| --- | --- | --- | --- |
| I am involved in the administration of vaccines to patients | 01 | 01 | **NURSES**  **MUST SELECT AT LEAST CODE 01 AND 02 AT S7a, AND S7b TO GO TO S8**  **Paediatricians and GPs/PCPs MUST SELECT AT LEAST CODE 02 OR 03 AT S7a**  **OTHERWISE THANK AND CLOSE** |
| I am responsible for discussing vaccine options and making recommendations to patients | 02 | 02 |  |
| I am involved in prescribing vaccines to patients | 03 | 03 |  |

S8 Which of the following vaccines have you administered, prescribed and/or recommended/personally discussed with [children/adolescents/adults] patients in the last 3 months?

**READ OUT, MULTICODE**

|  | | |
| --- | --- | --- |
| Influenza (Flu) | 1 – adult  1a: child/adolescent & adult [US only] | **ALL RESPONDENTS MUST SELECT AT LEAST CODE 5 OR 16 AND CODE 9a**  **OTHERWISE THANK & CLOSE**  **NURSE & GPs/PCPs: MUST SELECT AT LEAST 2 ADULT AND/OR TRAVEL VACCINES IN ADDITION [EXCEPT DE] TO CODE 5 AND CODE 9A**  **OTHERWISE THANK & CLOSE** |
| Hepatitis A vaccine | 2:child/adolescent 2a:adult |  |
| Hepatitis B vaccine | 3 child/adolescent 3a:adult |  |
| Herpes zoster (shingles) vaccine | 4 - adult |  |
| MMR (Measles, Mumps, Rubella) | 5 – child/adolescent |  |
| MMRV (Measles, Mumps, Rubella, Varicella) | 16 – child/adolescent |  |
| Meningococcal vaccine | 6 – child/adolescent |  |
| Pneumococcal vaccination | 7: child/adolescent 7a:adult |  |
| Tetanus, diphtheria, pertussis vaccine | 8 – child/adolescent |  |
| Diphtheria Tetanus, Polio Vaccine (DTP) / pentavalent / hexavalent | 9a – child/adolescent  9b: adult |  |
| Typhoid | 10 - Travel |  |
| Yellow fever | 11- Travel |  |
| Cholera | 12- Travel [except India]  12a: child/adolescent [in India only]  12b adult [in India only] |  |
| Human papillomavirus (HPV) vaccine | 13 – child/adolescent |  |
| Rabies | 14- Travel |  |
| Japanese encephalitis virus (JEV) | 15- Travel |  |

S9. In an average MONTH, approximately how many ADULT / ADOLESCENT / PEADIATRIC patients do you personally administer/prescribe and/or recommend/discuss vaccination ( not including flu season)

**WRITE IN NUMBER**

………………………………………number of adult patients [UK / US / DE]

………………………………………number of adolescent / paediatric patients [UK / US / DE / IN]

**GPs / PCPs:**

**UK – GO TO S10 IF MORE THAN 10 ADULT AND 10 ADOLESCENT/ PEADITRIC PATIENTS**

**DE – GO TO S10 IF MORE THAN 30 ADULT [PLEASE NOTE, NO QUOTA FOR ADOLESCENT/ PEADITRIC PATIENTS]**

**US – GO TO 0 IF MORE THAN 10 ADULT AND 20 ADOLESCENT/ PEADITRIC PATIENTS**

**NURSES:**

**UK – GO TO S10 IF MORE THAN 20 ADULT AND 60 ADOLESCENT/ PEADITRIC PATIENTS**

**US – GO TO S10 IF MORE THAN 20 ADULT AND 60 ADOLESCENT/ PEADITRIC PATIENTS**

**PAEDIATRICIANS ONLY:**

**DE – GO TO S10 IF MORE THAN 50 ADOLESCENT/ PEADITRIC PATIENTS**

**US – GO TO S10 IF MORE THAN 50 ADOLESCENT/ PEADITRIC PATIENTS**

**IN – GO TO S10 IF MORE THAN 50 ADOLESCENT/ PEADITRIC PATIENTS**

**OTHERWISE THANK & CLOSE**

S10. Which of the following best describes your attitude to vaccinations (in general)?

**SINGLE CODE**

|  | | |
| --- | --- | --- |
| I am a firm believer in the value of vaccinations | 1 | **Ensure a mix per group** |
| I believe in the value of vaccinations, however I don’t feel I get enough support | 2 |  |
| Vaccinations are part of my job | 3 |  |

S11 Have you taken part in any market research relating to vaccines in the last 1 month?

**SINGLE CODE**

| Yes | 01 | **THANK & CLOSE** |
| --- | --- | --- |
| No | 02 | **GO TO S11** |

S12 Are you, or any immediate family member, affiliated with any pharmaceutical company, healthcare manufacturer, or market research company?

**SINGLE CODE**

| Yes | 01 | **THANK & CLOSE** |
| --- | --- | --- |
| No | 02 | **GO TO S12** |

S13 The nature of the discussion group you are being asked to participate in will be very creative in nature: you will be asked to participate in activities that require you to think outside the box and/ or place yourself in/ act out hypothetical scenario(s). Do you feel comfortable in participating in activities such as these?

**SINGLE CODE**

| Yes | 01 | **GO TO D1** |
| --- | --- | --- |
| No | 02 | **THANK & CLOSE** |

D1 The interview may be observed by a video link and one way mirror by members of the sponsoring pharmaceutical company. Are you happy to participate with the interview on this basis?

**SINGLE CODE**

| Yes | 01 | **GO TO D2** |
| --- | --- | --- |
| No | 02 | **THANK & CLOSE** |

D2 We would like your consent for a short video recording of part of the discussion taken at the end of the discussion (the moderator will record this on a phone, and will inform you when they are about to start recording) to be passed to members of the Client Company sponsoring the research, on the condition that;

1. All use of information obtained from the interview is in accordance with the Data Protection Act
2. Your responses in the interview / audio recordings will be used solely in order to help members of the Company understand the research findings and for no other purpose. **Please be ensured that no sales approaches will ever be made to you as a consequence of the company having this access**
3. The people who will listen to the recording at the client company will be only in the following functions: market research, marketing, medical affairs and clinical development
4. All those hearing or seeing these recordings agree to abide by the Market Research Society, ABPI, and all other relevant (e.g. BHBIA, EphMRA and PBIRG) Codes of Conduct for market research
5. Your confidentiality and anonymity will be respected at all times and **no information can or will be directly attributed to you by name, nor will your name be made available to the sponsoring company, for any purpose**

Are you happy to give your consent to this at this point? You will also be re-asked for your consent at the end of the interview.

| Yes | 01 | **GO TO D3** |
| --- | --- | --- |
| No | 02 | **GO TO D3** |

**PLEASE NOTE RESPONDENT RESPONSE BUT YOU MAY RECRUIT/CONTINUE EVEN IF THEY SAY THEY DO NOT CONSENT TO RELEASE THE RECORDING TO THE SPONSORING COMPANY.**

D3 We are required to pass on to our client details of adverse events/product complaints pertaining to their products that are mentioned during the course of market research. Although what you say will, of course, be treated in confidence, should you raise during the discussion an adverse event or product complaint in a specific patient, or group of patients, we will need to report this even if it has already been reported by you directly to the company.

In such a situation you will be asked whether or not you are willing to waive the confidentiality given to you under the Market Research Codes of conduct specifically in relation to that adverse event/ product complaint. Everything else you say during the course of the interview will continue to remain confidential, and you will still have the option to remain anonymous if you so wish.

Are you happy to participate with the interview on this basis?

| Yes | 01 | **GO TO D4** |
| --- | --- | --- |
| No | 02 | **THANK & CLOSE** |

D4 Would you be happy for us to contact you again if we had any further clarifications after the interview? This would not mean another interview, it may be just a few points that we would appreciate and really value your opinion on further.

| Yes | 01 | **RECRUIT** |
| --- | --- | --- |
| No | 02 | **RECRUIT** |
