# Supplementary figures and images for: Challenges facing vaccinators in the 21st century: results from a focus group qualitative study
Source: Hum Vaccin Immunother. 2019 Jul 9;15(12):2806–15. doi: 10.1080/21645515.2019.1621147 (PMC6930098; doi:10.1080/21645515.2019.1621147)

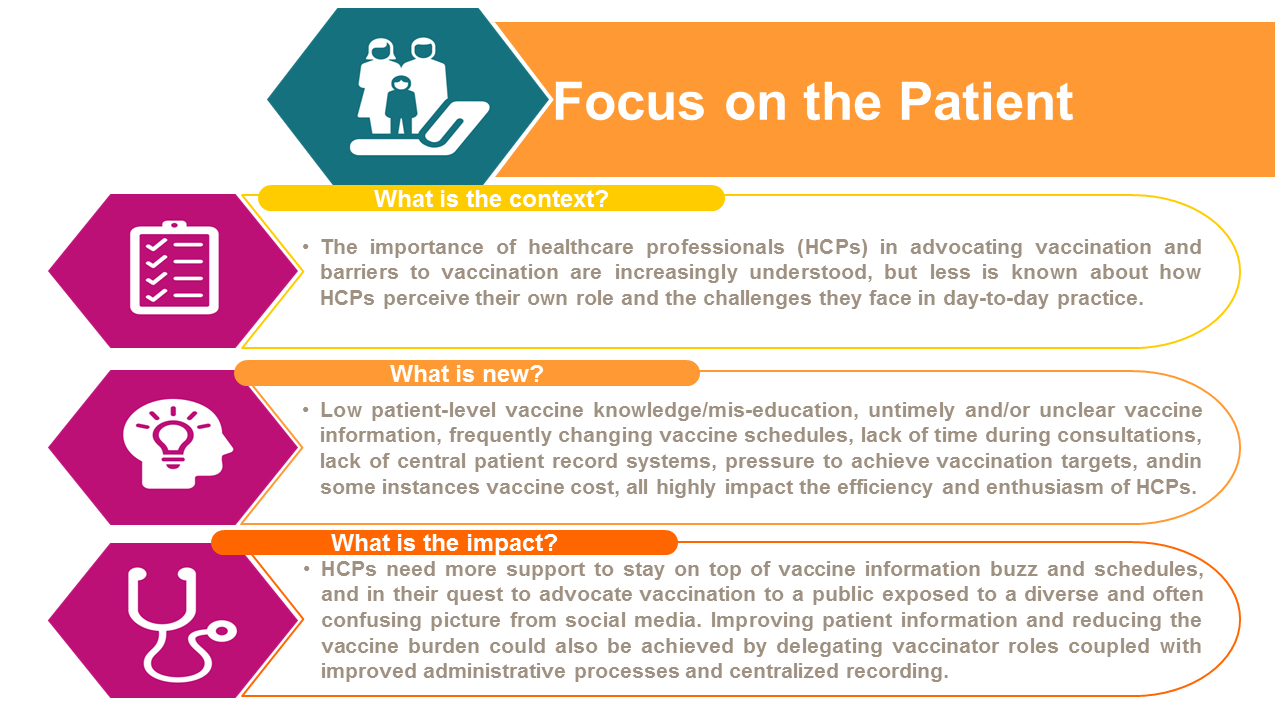

Supplement: Supplemental Material [file khvi-15-12-1621147-s001.zip › Truth about vaccinators_SuplApp1.TIF]
